# Gene Enrichment Analysis Reveals Major Regulators of *Mycobacterium tuberculosis* Gene Expression in Two Models of Antibiotic Tolerance

**DOI:** 10.3389/fmicb.2018.00610

**Published:** 2018-04-04

**Authors:** William M. Matern, Dalin Rifat, Joel S. Bader, Petros C. Karakousis

**Affiliations:** ^1^Department of Medicine, Johns Hopkins University School of Medicine, Baltimore, MD, United States; ^2^Department of Biomedical Engineering and High-Throughput Biology Center, Johns Hopkins University School of Medicine, Baltimore, MD, United States; ^3^Department of International Health, Johns Hopkins Bloomberg School of Public Health, Baltimore, MD, United States

**Keywords:** tuberculosis, regulon, enrichment analysis, transcription factors, antibiotic tolerance

## Abstract

The development of antibiotic tolerance is believed to be a major factor in the lengthy duration of current tuberculosis therapies. In the current study, we have modeled antibiotic tolerance *in vitro* by exposing *Mycobacterium tuberculosis* to two distinct stress conditions: progressive hypoxia and nutrient starvation [phosphate-buffered saline (PBS)]. We then studied the bacterial transcriptional response using RNA-seq and employed a bioinformatics approach to identify important transcriptional regulators, which was facilitated by a novel Regulon Enrichment Test (RET). A total of 17 transcription factor (TF) regulons were enriched in the hypoxia gene set and 16 regulons were enriched in the nutrient starvation, with 12 regulons enriched in both conditions. Using the same approach to analyze previously published gene expression datasets, we found that three *M. tuberculosis* regulons (Rv0023, SigH, and Crp) were commonly induced in both stress conditions and were also among the regulons enriched in our data. These regulators are worthy of further study to determine their potential role in the development and maintenance of antibiotic tolerance in *M. tuberculosis* following stress exposure.

## Introduction

*Mycobacterium tuberculosis* is the causative agent of tuberculosis (TB), which is the ninth most frequent cause of death worldwide, surpassing the mortality due to HIV/AIDS (World Health Organization, 2017). The success of *M. tuberculosis* as a human pathogen is related to its ability to persist in host tissues despite prolonged therapy with multiple antibiotics (World Health Organization, 2016). Several factors are believed to contribute to the phenomenon of antibiotic tolerance, which is defined as the reversible ability of bacteria to survive exposure to bactericidal antibiotics (Kester and Fortune, 2014; Brauner et al., 2016), including the harsh microenvironment encountered by *M. tuberculosis* bacilli within necrotic lung lesions (Dutta and Karakousis, 2014). Exposure of *M. tuberculosis* to progressive hypoxia and nutrient starvation *in vitro*, two stresses that are believed (Loebel et al., 1933; Via et al., 2008) to prevail within such lesions *in vivo*, results in bacterial growth arrest and tolerance to the cell wall-active agent isoniazid (Wayne and Hayes, 1996; Betts et al., 2002; Xie et al., 2005; Gengenbacher et al., 2010).

Prokaryotic cells can adapt and respond to environmental stresses by altering their gene expression or by modifying the activity or stability of existing proteins. Most commonly, bacterial metabolic adaptations are regulated at the transcriptional level through the action of transcription factors (TFs), which are DNA-binding proteins capable of repressing or activating transcription of specific genes (Browning and Busby, 2004). Examples of bacterial TFs include sigma factors, which confer promoter recognition and specificity to the RNA polymerase holoenzyme under various stress conditions (Ishihama, 2010), and two-component systems, usually consisting of a membrane-bound sensor histidine kinase and a cognate DNA-binding response regulator (Podgornaia and Laub, 2013). TFs exert their activity in the bacterial cell by activation of their respective regulons, which are a set of transcriptionally co-regulated operons (Liu et al., 2016). The number of TF genes present in each bacterial species appears to be proportional to the number of environmental stimuli encountered by that organism (Romero-Rodríguez et al., 2015). The *M. tuberculosis* genome is predicted to contain 209 different TF genes (Cole et al., 1998), attesting to the complicated lifecycle of the organism and the requirement for bacterial survival under different environmental stresses, including in airborne particles, along the epithelial lining of the airways, within the arrested phagosome of macrophages, and in the necrotic debris of caseous granulomas (Ehrt et al., 2015). Given the growing emergence of antibiotic resistance, the essentiality of bacterial transcription for prokaryotic cell viability under physiologically relevant stress conditions, and the lack of homology with human proteins, bacterial TFs represent an attractive target for drug development (Bem et al., 2015). An improved understanding of the TFs (and their respective regulons) required for *M. tuberculosis* survival during periods of stress may yield novel strategies to target persistent organisms, with the ultimate goal of shortening TB treatment.

In the current study, we used RNA-seq to study the change in global gene expression of *M. tuberculosis* during progressive hypoxia (Wayne and Hayes, 1996) and nutrient starvation (Betts et al., 2002), which induce bacterial stasis and tolerance to bactericidal antibiotics (Wayne and Hayes, 1996; Betts et al., 2002; Xie et al., 2005; Gengenbacher et al., 2010), relative to exponential growth in nutrient-rich broth. RT-qPCR was performed on a subset of genes to strengthen our confidence in the high-throughput data. A novel regulon enrichment test (RET) was used to identify enriched regulons corresponding to TFs in the *M. tuberculosis* stress response. Our efforts somewhat parallel the work of Du et al. (2016) but whereas these authors have focused on identifying regulators during reaeration (after hypoxia) we focus on core regulators of the response to multiple stresses. Our identified TFs may reflect potential vulnerabilities for the rational development of small molecule inhibitors targeting persistent *M. tuberculosis*.

## Materials and methods

### *In vitro* conditions

*Mycobacterium tuberculosis* strain CDC1551 (Ahmad et al., 2009) was used in all experiments. Prior to exposure to stress conditions, bacterial cultures were grown from frozen stocks to early exponential-phase (OD_600nm_ = 0.3) in Middlebrook 7H9 broth supplemented with 10% OADC enrichment (BD), 0.2% glycerol, and 0.05% Tween-80.

For nutrient starvation studies, the early exponential-phase bacteria were washed twice with phosphate-buffered saline (PBS) (Quality Biological Gaithersburg, MD) and resuspended in 100 ml of PBS containing 0.05% Tween-80 in a 250-ml flask. The final OD_600nm_ after resuspension was approximately 0.1. Cultures were incubated at 37°C for 3 days without shaking prior to RNA extraction. For progressive hypoxia studies, early exponential-phase bacteria were washed twice with Dubos-Tween-Albumin broth (DTA) and then resuspended in DTA containing 0.5 μg/mL methylene blue to a final OD_600nm_ of approximately 0.001. Twenty milliliters of this culture was added to 30-mL cylindrical glass tubes (19 × 145 mm) each containing a magnetic stir bar and plugged with a rubber stopper to prevent gas exchange. The tubes were then placed upright on a magnetic platform and checked every 2–3 days for color changes. Samples were collected when tubes were observed to have a yellowish color (about 14 days after inoculation).

### RNA extraction

At least 100 mL of bacterial culture (five hypoxia tubes or one PBS/Tween-filled flask) was used to generate sufficient material for a single RNA-seq sample. Bacteria were pelleted by centrifugation at 3,000 rpm at 4°C in 2-mL tubes containing 0.1 mm Zirconia/silica beads (BioSpec Products), and the cells were lysed by eight cycles of bead-beating for 30 s each (total of 4 min). The tubes were chilled on ice for 1 min after the first four cycles. The remainder of the extraction was performed as described previously (Thayil et al., 2011). At least three RNA samples were collected from each of the three conditions (7H9, PBS, or hypoxia). The quality of RNA samples was assessed using an Agilent Bioanalyzer (Agilent Technologies).

### RNA-seq

RNA-seq was performed by the Next Generation Sequencing Center at the Sidney Kimmel Comprehensive Cancer Center of the Johns Hopkins University School of Medicine. Strand-specific cDNA libraries were constructed using Encore® Complete Prokaryotic RNA-seq DR Multiplex Systems 1–8 and 9–16 (Nugen). Eight cDNA libraries were sequenced using an Illumina® HiSeq™ 2000.

### Identification of differentially expressed genes (DE)

Reads were de-multiplexed and adapter sequences removed to yield reads of 100-bp length. Raw reads were mapped to the *M. tuberculosis* CDC1551 genome (GenBank Assembly GCA_000669715.1) using EDGE-pro v1.3.1 (Magoc et al., 2013) (running Bowtie2 internally). Samples containing fewer than 5 × 10^5^ reads mapping to the CDC1551 genome were removed from the analysis (one sample) as these were assumed to suffer from technical errors during processing. After removal, our dataset included three samples from the log-phase condition, two from hypoxia, and two from PBS starvation. Output from EDGE-pro was input to DEseq2 v1.16.1 for differential expression analysis using default settings (Love et al., 2014). Fourteen additional RNA-seq samples (12 after removal of low-count samples), processed in an identical way, were also input to DESeq2 with our eight samples to improve the dispersion estimates. Briefly, these additional samples were collected from CDC1551 under a phosphate-depletion model (Rifat et al., 2009) and from a rpoB-H526D mutant (Rifat et al., 2017) (CDC1551 background) under the same set of stress conditions. Results from a careful analysis of these additional samples will be reported elsewhere. Genes were declared significantly differentially regulated if Benjamini–Hochberg adjusted *p*-values (Wright, 1992) were <0.05 and log2-fold-change relative to rich medium was >1 or <-1.

The GenBank flat file downloaded from NCBI the gene annotations labeled with a unique V735# and homologous Rv# (referencing the gene annotations of Mtb strain H37Rv). To allow for comparisons between this genome and the previously sequenced CDC1551 genome (Fleischmann et al., 2002), we built a local BLAST database of the CDC1551-2002 gene list and found the closest homologs in the new CDC1551 assembly using the command-line BLAST tool available from NCBI (ncbi-blast-2.4.0+ with settings: “blastn -task dc-megablast -outfmt 5 -evalue.001 -max_hsps 1 -max_target_seqs 50”). BioPython (Cock et al., 2009) v1.70 assisted with processing.

### Primer design and RT-qPCR

Primers for RT-qPCR were designed using Primer3 (Untergasser et al., 2012) followed by Primer-BLAST (Ye et al., 2012) to check for non-specific amplifications. Each designed primer was 18–20 nucleotides long and was predicted to yield a product of 95–105 nucleotides. *sigA* primers were designed previously, yielding a predicted 124-bp amplification product (Chuang et al., 2013). Primers were synthesized by Integrated DNA Technologies, Inc. A quick method to verify the efficiency of primer pairs was utilized. Given that all primer pairs were expected to yield a similar product size (and thus have similar fluorescence per molecule), we reasoned that all efficient pairs (>1.8 fold/cycle) should give approximately the same C_T_-value when genomic DNA was loaded in each reaction. This approach was used to test the C_T_-values for all primer pairs in triplicate. Primer pairs yielding products with significantly higher C_T_-values (>1.5 cycles from the median) were discarded and new primers were designed, as described above. The *sigA* primers, which were among the most highly efficient primers in our assay, were used for quality testing of redesigned primers. Additionally, melt curves were collected for all primer pairs using genomic DNA as template to confirm the absence of non-specific amplification. The primer pairs used for measuring expression of each gene are listed in Supplementary Data Sheet 6. Thirty-three genes were selected (in addition to *sigA*) for RT-qPCR follow-up among those genes found to be differentially expressed in the RNAseq data. Gene selection was non-random and were genes of interest to the investigators.

Total RNA (80–200 ng) was used for reverse transcription (RT) followed by qPCR. For each RNA sample, Ambion Turbo DNase (ThermoFisher) was used for genomic DNA removal. DNase was then inactivated by addition of EDTA (15 mM final concentration) and incubation at 75°C for 10 min. Each sample was aliquoted into two tubes. A freshly prepared RT master mix (Applied Biosystems High Capacity cDNA Reverse Transcription Kit, ThermoFisher) was added to one tube and the same master mix lacking RT enzyme was added to the other to serve as a no-RT control. Incubation and inactivation of the RT reactions were carried out according to the manufacturer's instructions. After RT, samples were diluted with a weak TE buffer (1.8 mM Tris-Cl, 0.18 mM EDTA, pH 8.0) to 1 mL total volume. qPCR was performed in a total well volume of 10 μL using 3 μL of each diluted sample per well (~120–300 pg of original RNA), 5 μL of 2x qPCR master mix (Biorad iTaq Universal SYBR Green Supermix), and each primer (5 pmol). Cycling conditions for qPCR were: 5 min at 95°C, followed by 40 cycles of [3 s at 95°C, 15 s at 55°C, and 30 s at 68°C]. Thermocycling was performed on an Applied Biosystems StepOnePlus qPCR machine using the fast ramp speed setting.

Technical triplicates were performed for each primer pair + sample. Additionally, no-RT and water controls (replacing sample cDNA with water) were included (in triplicate) on each qPCR plate. Primers for *sigA* were used to check for contamination in these controls. A cycle threshold (C_T_)-value for each well was computed using default settings from StepOne v2.2.2 software provided with the StepOnePlus machine. For analysis of differential expression, the median C_T_ of the three technical replicates was used throughout. The housekeeping gene *sigA* was used for normalization purposes and the delta-delta C_T_ method was used to quantify differential gene expression (Livak and Schmittgen, 2001). qPCR data was processed with custom Python3 scripts.

### Regulon enrichment test (RET) and mu-score

To find important regulators of the transcriptional responses we designed a novel RET. Our RET takes inspiration from the classic Fisher's Exact Test (FET), commonly used in gene enrichment analysis (Bian et al., 2017; Cheng et al., 2017), and is similar to the hypothesis test for large (2 × 2 and bigger) contingency tables considered by Agresti and Wackerly using Kendall's tau (Agresti and Wackerly, 1977) with the exception that our test does not penalize for certain off-diagonal entries. The authors of the VIPER and aREA algorithms consider a similar problem though their methods are significantly more sophisticated than ours (Alvarez et al., 2016).

Our RET operates on two sets of genes in which each gene has been labeled “up,” “down,” or “not-called.” This makes it amenable for use with many high-throughput data analysis pipelines where statistical methods are used to test the null hypothesis of no change in expression level against an alternative of change in one of two directions. In order to explain our method, let us call the two sets to be compared, sets A and B. In this study, we compared gene expression datasets collected from each stress condition (set A) with a transcription factor overexpression (TFOE) dataset (Turkarslan et al., 2015) (http://networks.systemsbiology.net/mtb/content/TFOE-Searchable-Data-File). Within the TFOE data, the changes in gene expression resulting from overexpression of each TF is set B. Therefore, the total number of RETs done (518) is equal to the number of stress conditions for which we collected gene expression data (2) multiplied by the number of TFs for which Turkarslan et al. collected data (209). In the TFOE dataset, for the overexpression of a particular TF, we declared genes “up” or “down” if the reported *p*-value for a gene is <0.01 and the log2-fold-change is >1 or <-1, respectively. Genes with larger *p*-values were declared “not-called.” These cutoff values were chosen based on the defaults provided in the spreadsheet provided by Turkarslan et al. In addition, we removed from the analysis those TFs where, upon induction with anhydrotetracycline, the authors observed a <0.5 log2-fold-change of the TF itself. Very slight changes in TF expression were assumed to unreliably induce the corresponding regulon. For each TF, 3–7 replicate microarrays were available in the TFOE dataset.

Our RET begins by forming a 3 × 3 contingency table comparing the two gene sets where the diagonal represents the number of genes found to be up, down, or not-called in both gene sets and the off-diagonal represents genes where the two datasets disagree (e.g., gene 1 is up in set A but down or not-called in set B). After table construction, we calculate an enrichment score (S) for how well the two datasets match:

$$\begin{array}{l} \text{S }=\;{\text{N}}_{\text{up},\text{up}}\;+\;{\text{N}}_{\text{down},\text{down}}\;-\left[{\text{N}}_{\text{up},\text{down}}\;+{\text{N}}_{\text{down},\text{up}}\right]. \end{array}$$

where N_up,up_ is the number of genes labeled as up in both datasets (and similarly for N_down,down_, N_up,down_, N_down,up_). Notably, genes not declared up or down in at least one of the datasets did not count toward the score, although these genes were retained in the analysis, as described below. This is advantageous as many datasets of interest have substantial numbers of genes in this category due to small sample sizes or strict statistical cutoffs, which can significantly affect the score if they are weighed positively or negatively.

To calculate the statistical significance of a 3 × 3 table we condition on the margins and assume that (at least) one of the datasets was generated randomly. Thus, a multivariate hypergeometric distribution is an appropriate model of randomness for the gene labeling (analogous to the univariate hypergeometric model assumed in enrichment analyses using the classic FET). One advantage to this setup over FET is that no categories need to be pooled or removed, thus preserving information and potentially increasing the power of the test.

Our chosen distribution can be described with an analogy. Imagine two persons playing a game. Each are given a list of genes and asked separately to place each gene into one of three categories: “up,” “down,” or “not-called” as they see fit. The players are told how many genes to include in each category. The score for the players is calculated as the number of genes both players placed into either the “up” or “down” category minus the number of genes the players placed in different categories. Any genes assigned to the “not-called” category by at least one player do not count toward the score. Assume that (at least) one of the players is assigning genes to categories by picking genes out of a hat. What is the probability that the score for the players is S or greater? This probability is the *p*-value. High scores (low *p*-values) suggest that neither player is picking categories out of a hat.

To calculate a *p*-value for an observed enrichment score ($\hat{\text{S}}$) we used Monte-Carlo sampling and a two-tailed hypothesis test [i.e., *p*-value = P(|S| > |$\hat{\text{S}}$|)]. Samples were generated from a multivariate hypergeometric distribution with parameters N_up,•_, N_down,•_, and N_nc,•_ (total number of genes labeled in dataset 1 as “up,” “down,” and “not-called,” respectively). The first N_•_,up (total number of genes labeled in dataset 2 as “up”) of these samples are labeled as “up” in Monte-Carlo simulated dataset 2 and so on for N_•_,down and N_•_,nc. Thus, the total number of sub-samples that must be drawn from the hypergeometric for a single Monte-Carlo sample is equal to the total number of genes considered (in our case, this is the number of genes in the CDC1551 genome). We generated 1 million Monte-Carlo samples for calculating each *p*-value.

Each Monte-Carlo computed *p*-value is only an estimate, and to be conservative we have used the one-sided Clopper-Pearson confidence interval to calculate a 95% upperbound for each *p*-value. Usually this adjustment makes only tiny changes in the *p*-value, but it can make a large difference when the region of rejection contains very few Monte-Carlo samples (such as for very small *p*-values, or small Monte-Carlo sample sizes).

After computation of *p*-values for each regulon we then use the Benjamini-Yekutieli (Benjamini and Yekutieli, 2001) procedure to control the False Discovery Rate at 25% (using the Python3 package, statsmodel v0.8.0 to compute the adjusted *p*-values). This method was chosen instead of the less-conservative Benjamini-Hochberg method as we are unable to prove that the set of TFOE regulons met the PRDS (Benjamini and Yekutieli, 2001) condition required for the correctness of the Benjamini-Hochberg procedure.

We also introduce a measure of the effect size for an enrichment, which we call the “mu-score.” The mu-score is defined as:

$$\begin{array}{l} \text{mu-score}=\text{S}/\left({\text{N}}_{\text{up},\text{up}}+{\text{N}}_{\text{down},\text{down}}+{\text{N}}_{\text{up},\text{down}}+{\text{N}}_{\text{down},\text{up}}\right). \end{array}$$

Therefore, the mu-score is bounded between −1 and +1, representing perfect discordance and perfect concordance, respectively. It is not defined if all genes are in the “not-called” category. We have found that the mu-score is an especially useful normalized metric when comparing with a TFOE dataset. In this case, the mu-score measures the down- or up-regulation of the TF regulon (as defined by the TF overexpression data). Specifically, a mu-score of +1 means that all genes induced/repressed under the condition studied were expressed in the same direction as during TF overexpression (discounting genes not differentially-expressed).

### Comparison with published microarray datasets

We compared our results with results published for a similar hypoxia model (Voskuil et al., 2004) and PBS starvation model (Betts et al., 2002). From the literature hypoxia dataset we used only the day 10 data for comparison as this was the time point where optical density reached its plateau (corresponding to Non-Replicating Persistence stage 2) (Wayne and Hayes, 1996). Wayne and Hayes (1996) report that the optical density plateau corresponds to dye discoloration and therefore we expected that data collected from this time point would be most comparable to our data. Following Voskuil et al., a gene was classified “up” or “down” if the intensity was at least 1.6-fold different compared to a log-phase culture (as specified for “moderately induced or repressed” transcripts). Otherwise the gene was declared “not-called.” Though experimental conditions were similar, there were some notable differences between the setup of Voskuil et al. and our own: they used Mtb strains H37Rv and clinical isolate 1,254 whereas we have used only strain CDC1551, they excluded methylene blue from their hypoxia cultures whereas we have included it, they used 2-color hybridizing microarrays to measure gene expression whereas we have used RNA-seq, for log-phase cultures they used a 7H9 medium excluding oleic acid and catalase whereas we have included these additives, and they started their hypoxia cultures at OD 0.004 whereas we have started ours at 0.001. Three technical replicates were available from this dataset.

From the published PBS starvation model data (Betts et al., 2002) we used the 96 h time point as this was the closest available (in h) to our sampling time point at 72 h. We used the list of differentially regulated genes as provided in their supplement data (genes not on this list were considered “not-called”). Though experimental conditions were similar, there were some notable differences between the setup of Betts et al. and our own: for log-phase cultures they used a 7H9 medium excluding oleic acid whereas we have included this additive, and for PBS starvation cultures they excluded Tween-80 whereas we have included it to reduce bacterial clumping. Three replicates were available from this dataset.

We applied our RET to find enriched regulons in each of the literature datasets using the same TF overexpression dataset (Turkarslan et al., 2015). We used the same cutoff for adjusted *p*-values to define enriched regulons.

### Distribution of code and datasets

Code to replicate the performed analysis (except for analysis of the qPCR data) is available as a repository on GitHub (https://doi.org/10.5281/zenodo.1013210) under the MIT license. Code was written in a combination of R, Python3, bash, and makefile. The raw reads and gene expression summary data are available in the Gene Expression Omnibus (GSE104599). Note that the raw data for the 14 additional samples added to improve the dispersion estimate of Deseq2 are included in the GEO submission. However, summary data for these additional samples are not provided as this analysis is ongoing.

## Results

RNA-seq revealed the upregulation of a total of 462 and 337 *M. tuberculosis* genes (with 185 genes in the intersection) during progressive hypoxia and nutrient starvation, respectively, relative to exponential growth. Conversely, 490 and 262 genes (153 genes in the intersection) were downregulated during *M. tuberculosis* exposure to progressive hypoxia and nutrient starvation, respectively (Supplementary Data Sheet 1).

**Figure 1:**
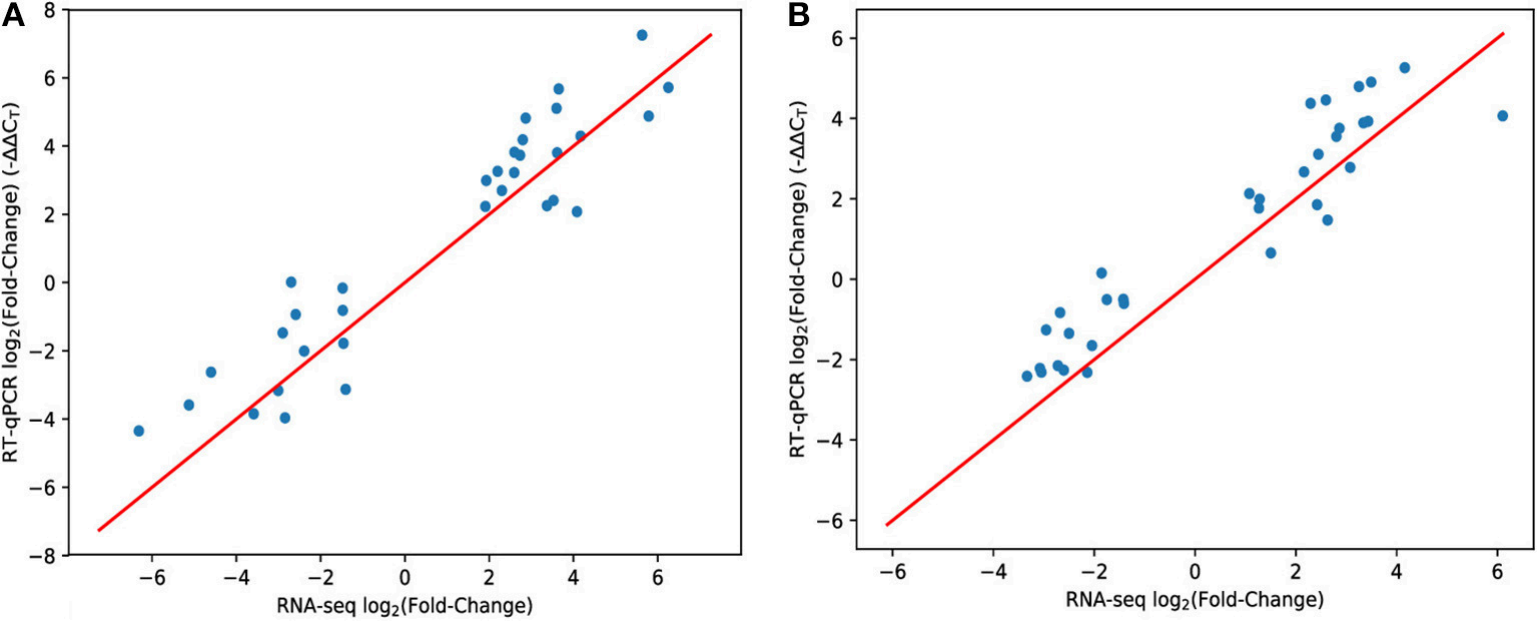
Comparison of RNA-seq and RT-qPCR methodologies. Each data point represents a single gene. The x-axis shows the mean expression fold change of each gene in hypoxia **(A)** or PBS nutrient starvation **(B)** relative to 7H9 using the RNA-seq methodology (and inferred with DESeq2). The y-axis is the same measurement (mean ΔΔ*C*_*T*_) but using the RT-qPCR methodology to measure gene expression. The red line is y = x. Genes were hand-selected based on the RNA-seq data.

Since high-throughput techniques such as RNA-seq may be prone to systematic variation and biases, we tested a subset of genes by RT-qPCR using the same experimental samples. Overall, RT-qPCR yielded similar results to RNA-seq (Figure 1). A few genes showed somewhat reduced magnitudes of differential expression using RT-qPCR but the overall trend is strong (*R*^2^ = 85% for hypoxia, *R*^2^ = 81% for nutrient starvation—using the *y* = *x* model to compute the residual sum of squares).

In order to identify TFs that may play an important role in *M. tuberculosis* adaptation to progressive hypoxia and nutrient starvation, we used the large dataset published by Turkarslan et al. (2015), in which the authors measured global *M. tuberculosis* gene expression following overexpression of 209 different TFs, and applied our RET to our RNA-seq data from each stress condition. We found that 17 TF regulons were enriched in the hypoxia gene set and 16 TF regulons were enriched in the nutrient starvation dataset (Supplementary Data Sheets 2, 3). A total of 12 TF regulons were enriched in both conditions (Table 1). The induction of 4 regulons was accompanied by concomitant upregulation of the corresponding TF (*sigE, whiB3*, and *sigH, Rv1049*). However, five regulons were upregulated in each stress condition while their corresponding TFs were significantly downregulated (*whiB5, Rv0023, Rv0757, Rv0818*, and *sigC*), and the downregulation of one regulon was accompanied by significantly increased expression of its TF (*Rv2887*). The remaining two TFs (*Crp, Rv0081*) were not expressed in the same direction under each of the stresses though their regulons were upregulated (mu-score > 0). Notably, in both stress models and both datasets, the regulons were expressed in the same direction (ie mu-score was always either positive or negative).

**Table 1:** Regulons showing significant enrichment using our collected RNA-seq data.

**TF name**	**Hypoxia mu-score**	**Hypoxia TF log2FC**	**PBS mu-score**	**PBS TF log2FC**	**TF Description (NCBI)**
Rv0022c	0.63	−1.78	0.57	−0.94	Transcriptional regulator WhiB-like WhiB5
Rv0023	0.50	−3.23	0.74	−1.55	Transcriptional regulator
Rv0081	0.50	0.85	0.66	−1.96	ArsR family transcriptional regulator
Rv0757	0.72	−0.10	1.00	−0.31	OmpR family two-component system response regulator
Rv0818	0.58	−1.12	0.62	−1.09	Transcriptional regulator
Rv1049	0.79	0.79	0.65	1.48	Transcriptional repressor
Rv1221	1.00	3.65	1.00	3.49	RNA polymerase sigma factor SigE
Rv2069	1.00	−0.84	0.75	−0.07	RNA polymerase sigma factor SigC
Rv2887	−0.71	2.20	−0.84	1.33	Transcriptional regulator
Rv3223c	0.57	2.73	0.46	1.27	RNA polymerase sigma factor SigH
Rv3416	0.44	2.20	0.56	2.80	Transcriptional regulator WhiB-like WhiB3
Rv3676	0.78	0.04	0.75	−0.41	Transcriptional regulator Crp

*For each dataset, mu-scores are as defined in the Methods section. log2FC = log (base 2) of the fold change in read counts for the TF itself relative to log-phase growth*.

We next applied our RET to previously published microarray datasets describing *M. tuberculosis* transcriptome changes following exposure to progressive hypoxia (Voskuil et al., 2004) and nutrient starvation (Betts et al., 2002). Twelve regulons, including the DosR regulon, were enriched in the previously published hypoxia dataset, and 19 regulons were enriched in the nutrient-starvation dataset (Supplementary Data Sheets 4, 5). Three regulons were commonly enriched in both conditions (Table 2). All three were also found to be enriched in the same directions in our RNA-seq data (Table 1).

**Table 2:** Regulons showing significant enrichment in two previously published gene expression datasets collected from a hypoxia and a PBS-starvation model.

**TF name**	**Hypoxia mu-score**	**Hypoxia TF log2FC^1^**	**PBS mu-score**	**PBS TF log2FC^2^**	**TF description (NCBI)**
Rv0023	0.64	−0.55	0.66	−0.73	Transcriptional regulator
Rv3223c	0.62	0.59	0.53	−0.33	RNA polymerase sigma factor SigH
Rv3676	0.47	−0.47	0.60	0.21	Transcriptional regulator Crp

mu-scores are as defined in the Methods section. log2FC = log (base 2) of the fold change in fluorescence for the TF itself relative to log-phase growth.

1 Data from Voskuil et al. (2004);

2 *Data from Betts et al. (2002)*.

## Discussion

Recently, there has been significant interest in targeting phenotypically tolerant *M. tuberculosis* to shorten anti-TB therapy (Gold and Nathan, 2017). However, the molecular mechanisms underlying mycobacterial antibiotic tolerance remain to be elucidated (Gold and Nathan, 2017). An improved understanding of the regulatory pathways governing antibiotic tolerance in *M. tuberculosis* may yield novel strategies to accelerate eradication of infection. In particular, *M. tuberculosis* TFs regulating antibiotic tolerance during infection-relevant stress conditions may represent attractive targets for drug development, as it may be possible to disturb TF-DNA binding and inhibit the tolerant state with sufficient specificity to avoid human toxicity. As proof of concept, the novel compound SMARt-420 was recently shown to sensitize *M. tuberculosis* to ethionamide by inhibition of a transcriptional repressor (Rv0078) of an ethionamide-activating enzyme (Rv0077c) (Blondiaux et al., 2017). Additionally, elucidation of *M. tuberculosis* stress adaptation strategies may yield insight into the selective susceptibility of the organism to the key sterilizing drug pyrazinamide during anaerobic incubation (Wade and Zhang, 2004).

In this study, we have identified several TFs and regulons of potential interest in the adaptation of *M. tuberculosis* to hypoxia and nutrient starvation. Perhaps most intriguing are those regulons responding to both stresses (Table 1), as these may represent important components of a universal *M. tuberculosis* stress response. We speculate that at least some of these regulatory pathways may be involved in the development of antibiotic tolerance, which is a common phenotypic feature of *M. tuberculosis* exposed to each condition.

There are several potential explanations for the discordance we observed between mu-scores and log_2_-fold-change for certain TF/regulon pairs (i.e., different expression directions for a TF and its corresponding regulon). The regulons with large mu-scores but with no change in or opposite changes in the corresponding TF transcript levels may undergo a post-translational modification, becoming more (or less, in the case of *Rv2887*) active during the stress condition relative to nutrient-rich broth. Well-described mechanisms for changes in the activation status of TFs include undergoing a chemical/conformational change [e.g., phosphorylation (Roberts et al., 2004) or binding of ligands (Pyles and Lee, 1996)] or a change in the activation of protein partners (Song et al., 2003). In fact, this hypothesis has been described in the case of CRP_MT_ (Rv3676), as discussed below. Another possible explanation for these discrepancies is the potential loss of correlation between transcript levels and protein levels due to heterogeneity in protein degradation rates or because transcriptional changes precede changes in protein levels.

The gene *rv3676*, also known as *crp* [cyclic adenosine monophosphate (cAMP)-responsive protein], is known to encode a TF (CRP_MT_) that binds cAMP. We found that its regulon was upregulated (positive mu-score) in both our RNA-seq data and in the previously published microarray datasets under both hypoxia and nutrient starvation conditions (Tables 1, 2). Previous studies have highlighted the considerable overlap between CRP_MT_-regulated genes and the *M. tuberculosis* transcriptional responses to hypoxia and PBS (Bai et al., 2005). While CRP_MT_ has some activity without bound cAMP, this ligand increases the affinity of CRP_MT_ for DNA (Bai et al., 2005). This finding may explain our observation that CRP_MT_ transcript levels are similar or downregulated under the two stress conditions studied while the CRP_MT_ regulon is upregulated. It has also been noted that many of the known adenylate cyclases (ACs), which synthesize cAMP, are upregulated during hypoxia and PBS starvation (Bai et al., 2011). Though cAMP levels do not appear to have been measured in *M. tuberculosis* during hypoxia or nutrient starvation, our results would fit well with a model where hypoxia and nutrient starvation trigger an increase in the levels of cAMP (possibly through upregulation of ACs) leading to increased activity of CRP_MT_ and expression of its regulon. *In vivo*, an *M. tuberculosis* recombinant strain deficient in CRP_MT_ was shown to have a significant growth defect in the lungs and spleens of BALB/c mice (Rickman et al., 2005).

The SigH (Sigma factor H) regulon was consistently upregulated (positive mu-score) in our RNA-seq data and in the previously published microarray datasets under both hypoxia and nutrient starvation conditions (Tables 1, 2). However, *sigH* itself was found to be slightly downregulated by Betts et al. (2002) during nutrient starvation, yet upregulated in the other three datasets. It is possible that differences in the experimental setups could account for the discrepancy with the Betts et al. dataset (see Methods section for a summary of these). Additionally, the microarrays used by Betts et al. (2002) and Voskuil et al. (2004) lacked a probe for the cognate anti-sigma factor H gene, *rshA* (*rv3221A*), which obscures the role this anti-sigma factor may have played in the observed activation of the SigH regulon. SigH activation has been shown to increase expression of several genes, including genes involved in maintaining redox equilibrium and in protein degradation. *sigH* was shown to be dispensable for *M. tuberculosis* growth in the lungs of C57BL/6 and C3H mice, but was required for lung immunopathology and lethality (Kaushal et al., 2002). On the other hand, an *M. tuberculosis* recombinant strain deficient in *sigH* was found to be defective for survival in the lungs of non-human primates and also induced less immunopathology (Mehra et al., 2012).

The Rv0023 regulon was also upregulated (positive mu-score) in both our RNA-seq data and in the previously published microarray datasets under both hypoxia and nutrient starvation conditions (Tables 1, 2). Expression of *rv0023* was consistently downregulated across the various stress conditions and in the previously published datasets. Thus, Rv0023 may undergo an increase in activation status during the transition from exponential growth in nutrient-rich broth to growth-limiting conditions. Rv0023 is not well-studied but is known to be involved in the *M. tuberculosis* response to hypoxia (Rustad et al., 2014). Our data also support a role for this protein in the *M. tuberculosis* response to nutrient starvation. The *rv0023* gene has been predicted to be non-essential, although deficiency of this gene appears to induce a growth defect (DeJesus et al., 2017). Future studies to evaluate the role of *rv0023* in *M. tuberculosis* antibiotic tolerance and survival during stress conditions will likely require extended incubation to cultivate a genetic deletion strain and/or the use of conditional knock-down systems (Kim et al., 2013; Rock et al., 2017).

Additionally, we found nine regulons (WhiB5, Rv0081, Rv0757, Rv0818, Rv1049, SigE, SigC, Rv2887, WhiB3) to be significantly enriched in our nutrient starvation and hypoxia models, although they were not significantly induced in at least one of the literature datasets. Rv0757 (PhoP) is a two-component system regulator, which is phosphorylated by PhoR (a histidine kinase) (Ryndak et al., 2008). It has previously been linked to the *M. tuberculosis* hypoxia response (Galagan et al., 2013), and our data suggests it is also activated during nutrient starvation. Rv0818 (GlnR) has been previously linked to nitrogen starvation. Our results suggest GlnR is also activated during progressive hypoxia. The Rv2887 regulon was the only one found to be downregulated despite increased transcript levels of the TF during each of the stress conditions, suggesting inactivation of Rv2887 during stress exposure. Loss-of-function mutants in this TF have previously been associated with resistance to novel antimycobacterials (Winglee et al., 2015; Warrier et al., 2016), but not to the first line anti-mycobacterial agents isoniazid and rifampicin, as measured by MIC. However, it remains to be determined whether *rv2887* deficiency is associated with *M. tuberculosis* antibiotic tolerance, as measured by minimum bactericidal concentration or time-kill kinetics.

Somewhat surprisingly, we did not identify the DosR regulon (as defined by the Turkarslan et al. data) as enriched (in either direction) in our RNA-seq data (Supplementary Data Sheet 2). However, the DosR regulon was detected as significantly enriched in the previously published microarray data using our RET (Supplementary Data Sheet 4). Therefore, it appears the DosR regulon was simply not expressed in our hypoxia model. These discrepant findings may be attributable to methodological differences between our study and that of Voskuil et al. (2004), including the use of methylene blue in our study and the different bacterial strains used in each study. Other potential contributing factors are described in the Materials and Methods. Despite this, we found that all the regulons enriched in both the published hypoxia dataset and the nutrient starvation datasets (Rv0023, SigH, and Crp) were also enriched in our analysis, underscoring the similarities in experimental design between the previously published studies and ours.

This study had several limitations. The TF overexpression dataset we used (Turkarslan et al., 2015) may yield false positive data since these genes may have been overexpressed at superphysiological levels, leading to non-specific DNA binding and transcription, or may have triggered a non-specific stress response unrelated to the specific TF in question. Furthermore, given that the TF overexpression dataset was collected only in nutrient-rich broth, it is possible that we missed additional TFs, which are only active under stress conditions. Notably, 62 (of the 209) regulons had 2 or fewer members suggesting these TFs may have limited activity in nutrient-rich conditions. Additionally, while oxygen is limited within the tuberculous lesions where at least some bacilli are believed to reside *in vivo* (Via et al., 2008; Hoff et al., 2011), broad nutrient starvation models have been argued to simulate lack of some particular molecule that is necessary for bacterial growth or virulence (Loebel et al., 1933). However, a precise description of the factors that are lacking is vaguely defined in the literature and a thorough cataloging effort to determine the levels of available nutrients within these lesions has yet to be undertaken, although a recent effort suggests this may be possible with existing technology (Marakalala et al., 2016).

With respect to the bioinformatics analysis, no null-model of gene enrichment completely captures the true distributions involved. Our choice assumes that at least one of the datasets is “random” with fixed numbers of genes in each category, thus motivating our use of the multivariate hypergeometric. Another approach would be to allow the number of genes in each category to vary (i.e., a multinomial distribution) and compute the maximum *p*-value over all possible multinomial parameters, analogous to Barnard's test for 2D tables (Barnard, 1947). However, this likely becomes computationally difficult with the 3 × 3 tables considered here. More sophisticated models could include prior knowledge of gene regulation, though defining the relevant statistical distributions to perform a hypothesis test may require significant data gathering. Additionally, the Monte-Carlo method we have used here to compute *p*-values for the RET is computationally slow, suggesting another area for improvement of this method possibly by modifying existing exact methods (Mehta and Patel, 1983).

Our study highlights some specific *M. tuberculosis* TFs that we believe are worthy of further study. Recombinant strains deficient in these TFs should be tested for survival during exposure to hypoxia, nutrient starvation, and other physiologically relevant stress conditions. In addition, such mutants should be tested for susceptibility to bactericidal antibiotics, such as isoniazid, under these conditions to examine the role of the missing TF/regulon in observed antibiotic tolerance.

## Author contributions

DR and PK planned the study. DR performed the experimental studies. WM performed bioinformatics and statistical analysis, contributed to the experimental studies, and wrote the manuscript. JB and PK edited the manuscript. PK provided material resources.

### Conflict of interest statement

The authors declare that the research was conducted in the absence of any commercial or financial relationships that could be construed as a potential conflict of interest.
